# Retinoids: present role and future potential

**DOI:** 10.1038/sj.bjc.6690312

**Published:** 1999-04

**Authors:** T R J Evans, S B Kaye

**Affiliations:** CRC Department of Medical Oncology, University of Glasgow, Alexander Stone Building, Garscube Estate, Switchback Road, Bearsden, Glasgow G61 1BD, UK

**Keywords:** differentiation, apoptosis, drug resistance, retinoids

## Abstract

Vitamin A and its biologically active derivatives, retinal and retinoic acid (RA), together with a large repertoire of synthetic analogues are collectively referred to as retinoids. Naturally occurring retinoids regulate the growth and differentiation of a wide variety of cell types and play a crucial role in the physiology of vision and as morphogenic agents during embryonic development. Retinoids and their analogues have been evaluated as chemoprevention agents, and also in the management of acute promyelocytic leukaemia. Retinoids exert most of their effects by binding to specific receptors and modulating gene expression. The development of new active retinoids and the identification of two distinct families of retinoid receptors has led to an increased understanding of the cellular effects of activation of these receptors. In this article we review the use of retinoids in chemoprevention strategies, discuss the cellular consequences of activated retinoid receptors, and speculate on how our increasing understanding of retinoid-induced signalling pathways may contribute to future therapeutic strategies in the management of malignant disease. © 1999 Cancer Research Campaign


*British Journal of Cancer* (1999) **80**(1/2), 1–8
© 1999 Cancer Research Campaign
Article no. bjoc.1998.0312

### Retinoids: present role and future potential

**TRJ Evans and SB Kaye**

CRC Department of Medical Oncology, University of Glasgow, Alexander Stone Building, Garscube Estate, Switchback Road, Bearsden, Glasgow G61 1BD, UK

**Summary** Vitamin A and its biologically active derivatives, retinal and retinoic acid (RA), together with a large repertoire of synthetic analogues are collectively referred to as retinoids. Naturally occurring retinoids regulate the growth and differentiation of a wide variety of cell types and play a crucial role in the physiology of vision and as morphogenic agents during embryonic development. Retinoids and their analogues have been evaluated as chemoprevention agents, and also in the management of acute promyelocytic leukaemia. Retinoids exert most of their effects by binding to specific receptors and modulating gene expression. The development of new active retinoids and the identification of two distinct families of retinoid receptors has led to an increased understanding of the cellular effects of activation of these receptors. In this article we review the use of retinoids in chemoprevention strategies, discuss the cellular consequences of activated retinoid receptors, and speculate on how our increasing understanding of retinoid-induced signalling pathways may contribute to future therapeutic strategies in the management of malignant disease.

**Keywords:** differentiation; apoptosis; drug resistance; retinoids

### RETINOIDS AND THEIR RECEPTORS

### Chemoprevention

Many cancers develop as a result of exposure to carcinogens and cancer-promoting agents in a multistep process including both initiation and promotion. Attempts to delay, reverse or block cancer development by intervening in this process form the basis of cancer chemoprevention strategies. An increased susceptibility to chemical carcinogens and a higher incidence of cancer have been observed in experimental animals with vitamin A deficiency (Moon et al, 1994). This, and the observation that individuals with a lower dietary intake of vitamin A are at a higher risk of developing cancer (Hong and Itri, 1994), gave rise to the notion that physiological levels of retinoids may, in some way, protect the individual against the development of premalignant and malignant disease. Furthermore, the efficacy of pharmacological doses of retinoids as chemopreventive agents has been demonstrated in experimental models of carcinogenesis for numerous animal tumours (reviewed in Lotan, 1996).

#### *Preclinical data*

Retinoids can inhibit the transformation of cultured mouse embryo cells in vitro by either 3-methylcholanthrene (Bertram, 1983) or γ-rays. Retinoids can also inhibit the ability of malignant cells to form colonies in semisolid medium, an anchorage-independent property that is a characteristic of transformed cells (Lotan, 1995). Moreover, all-*trans* retinoic acid (ATRA) can inhibit immortalization of human epidermal keratinocytes during or after transfection with HPV16 (Creek et al, 1994). In addition, the synthetic retinoid *N*-(4-hydroxyphenyl)retinamide (4HPR) can inhibit prolactin-induced DNA synthesis and end-bud proliferation in mouse mammary gland in whole organ culture (Moon et al, 1994), and retinoids can also modulate normal rat mammary epithelial cell proliferation, morphogenesis and functional differentiation (Lee et al, 1995).

The use of retinoids to suppress tumour development has been evaluated in several animal models of carcinogenesis including models of skin, breast, oral cavity, lung, hepatic, gastrointestinal, prostatic and bladder cancers (reviewed in Lotan, 1996). Many of these studies have shown that retinoids possess antipromotion activities. However, long-term retinoid treatment was required to suppress carcinogenesis since the effects of the retinoids were reversible when stopped. Furthermore, some retinoids were found to be active in certain animal models of carcinogenesis and not in others, that is retinoids exhibit some degree of tissue selectivity. Moreover, certain retinoids may be active inhibitors of carcinogenesis in certain tissues but can act as enhancers of carcinogenesis in the same tissue in another strain of mice, or in another carcinogenesis model. For example, dietary addition of ATRA had no effect on tumour initiation in the two-stage mouse skin carcinogenesis model, but acted as an antipromoter by inhibiting progression of papilloma to carcinoma (De Luca et al, 1994). However, retinoids were either ineffective in preventing, or enhanced, papilloma formation when an alternate mouse skin carcinogenesis model was used (De Luca et al, 1994). Indeed, vitamin A deficiency was more effective than excess retinoid in inhibiting skin carcinogenesis using an alternative strain of mice (Lotan, 1996). Similarly, both suppression and enhancement by retinoids have been reported in different models of liver carcinogenesis (Moon et al, 1994). Indeed, 4HPR suppressed carcinogenesis in two strains of mice and enhanced carcinogenesis in two other strains (Moon et al, 1994). Inconsistent results were also obtained in models of lung, oesophageal and pancreatic carcinogenesis (Moon et al, 1994). Studies using rat mammary models have confirmed

*Received 17 March 1998*
*Revised 19 November 1998*
*Accepted 4 December 1998*

*Correspondence to:* TRJ Evans the importance of tissue distribution and metabolism of retinoids. Retinoids that accumulated in the mammary gland and the surrounding fat pad were more effective inhibitors of carcinogenesis than those that failed to concentrate in the target tissue. Again, retinoids were ineffective as inhibitors of initiation in these rat mammary models but, interestingly, administration of retinoid in early phases of carcinogenesis resulted in sustained inhibition of carcinogenesis even after stopping retinoid treatment, whereas a delay in retinoid administration until later in the carcinogenic process required continuous retinoid administration to maintain an inhibition of carcinogenesis. In contrast, pre-treatment of rats with retinoids for 2 months prior to initiation resulted in an increased incidence of carcinomas unless retinoid administration was continued through to the promotion step (reviewed in Moon et al, 1994; Lotan, 1996). Consequently, understanding the timing of administration in addition to tissue distribution and metabolism is crucial if retinoids are to be effectively used as chemopreventive agents.

### *Clinical data*

Many clinical trials of retinoids as chemoprevention agents are in progress or have been completed. Most of these trials focus on individuals at an increased risk of developing cancer, such as patients with pre-malignant lesions or patients who have been successfully treated for an early-stage carcinoma and have a high risk of developing a second primary cancer. The use of retinoids in patients with cutaneous actinic keratoses results in a significant decrease in the incidence of squamous cell carcinomas of the skin (Moon et al, 1997), and this is also observed in renal transplant patients with this pre-malignant condition (Bavinck et al, 1995; Rook et al, 1995). Similarly, topical ATRA can lead to a clinical and histological improvement in patients with the dysplastic nervous syndrome (Edwards and Jaffe, 1990; Halpern et al, 1994). Oral pre-malignant lesions such as leukoplakia or erythroplakia are frequently extensive or multiple and consequently are not amenable to surgery. Therefore, patients with these lesions are ideal candidates for chemoprevention studies. In an original placebo-controlled randomized study, 13-*cis* retinoic acid was evaluated (Hong et al, 1986). Although there was a significant clinical response in the treatment arm (67%) compared to the placebo arm (10%), the treatment was unacceptably toxic and half of the responding patients had relapsed within 3 months of stopping the retinoid (Hong et al, 1986). Subsequently, patients received this dose for only a 3-month induction period, and were then randomized to either low-dose retinoid or β-carotene for 9 months (Lippman et al, 1993, 1995). This study demonstrated the feasibility of using low-dose 13-*cis* retinoid acid in maintaining initial responses, whilst underlining the inability of β-carotene to do so. Furthermore, topical 4HPR also shows promising activity in the management of pre-malignant oral lesions (Chiesa et al, 1993; Costa et al, 1994). Cancer of the cervix, which develops in a multistep fashion through progressive intra-epithelial neoplasia (CINI–III) is another logical candidate for a chemoprevention approach. Topical application of ATRA-induced regression in 43% of patients with moderate dysplasia (CIN II) in comparison to a spontaneous regression in 27% of patients treated with placebo, but had no effect on more severe dysplasia (Meyskens et al, 1994). Moreover, *N*-(4-ethoxycarbophenyl) retinamide decreased the incidence and increased survival in a population at high risk of oesophageal cancer (Han, 1993), but retinoids have had consistently no effect on reversal of bronchial metaplasia (Lee et al, 1994).

An unexpected observation from the adjuvant studies of 13-*cis* retinoic acid in early stage head and neck cancers was the reduction in incidence of second primary tumours, although there was no reduction in the rate of recurrence or metastases from the original primary (Hong et al, 1990; Benner et al, 1994). In contrast, etretinate was ineffective in preventing second primary tumours of the oral cavity and oropharynx (Bolla et al, 1994). Similarly, retinyl palmitate can reduce the incidence of second primary tumours in comparison to observation after resection of stage I non-small cell lung cancer (Pastorino et al, 1993). Moreover, initial studies evaluating 13-*cis* retinoic acid in the prevention of recurrent early-stage bladder cancer had to be terminated due to toxicity and a lack of positive results (Prout and Barton, 1992), but etretinate in contrast can increase the time to recurrence in patients with superficial papillary bladder cancers with a reduction in the number of annual transurethral resections (Studer et al, 1995). Based on the preclinical data, a randomized study comparing 4HPR with observation alone has been initiated in women with node-negative breast cancer, and accrual to this study has been completed (Costa et al, 1995). The end-point is the incidence of contralateral primary carcinoma, and clearly if 4HPR can reduce this risk (estimated at 0.8% per year within 10 years of primary treatment), then it would be appropriate to evaluate this agent as a chemoprevention strategy in women at high risk of developing breast cancer on the basis of family history.

However, the most prominent example of the role of retinoids as differentiating agents in current oncology practice is the remarkable activity of all-*trans* retinoic acid in patients with acute promyelocytic leukaemia (APL). Numerous phase II studies have confirmed that ATRA induces complete remission in the vast majority of patients, with rapid resolution of the characteristic, life-threatening coagulopathy (Huang et al, 1988; Castaigne et al, 1990; Chen et al, 1991; Warrell et al, 1991; Fenaux et al, 1992; Ohno et al, 1993; Frankel et al, 1994; Kanamaru et al, 1995). The duration of complete remission with ATRA alone is usually brief and post-remission chemotherapy is required to diminish the likelihood of relapse. A randomized study has confirmed that ATRA as induction or maintenance treatment improves disease-free and overall survival as compared with chemotherapy alone, and should be included in the treatment of APL (Tallman et al, 1997). This therapeutic approach has also contributed to our understanding of retinoid-induced signalling pathways and in particular the role of the retinoic acid receptors.

### The retinoid receptors

Retinoids exert most of their effects by binding to specific receptors and modulating gene expression. The nuclear retinoid receptors are members of the steroid/thyroid hormone superfamily of receptors (Evans, 1988) with which they share common structural and functional properties. The diversity of retinoid-induced signalling pathways is mediated by at least six retinoid receptors which fall into two subfamilies: retinoic acid receptors (RARs), α, β and γ, and the retinoid X receptors (RXRs), α, β and γ (Chambon, 1995). In common with other members of the steroid hormone receptor superfamily, these two subfamilies of receptors contain a DNA-binding domain and a ligand-binding domain united by a short hinge region that may also serve as a nuclear

© Cancer Research Campaign 1999

translocation signal (reviewed in Giguere, 1994). The DNA-binding domain contains two 'zinc fingers' involved in recognition of specific DNA sequences and in activation of target genes (Evans, 1988; Freedman, 1992). The RARs bind ATRA with high affinity (Giguere et al, 1987; Petkovich et al, 1987), whereas the stereoisomer 9-*cis* retinoic acid is a bifunctional ligand which can bind to and activate both RARs and RXRs (Mangelsdorf et al, 1992; Allenby et al, 1993). Despite these similarities, the RXRs belong to a subgroup of nuclear receptors distinct from the RARs (Laudet et al, 1992) suggesting that these two groups of retinoid receptors have distinct roles in retinoid signalling.

In keeping with the models by which steroid hormone receptors bind DNA as dimers, the hormone response elements of the target genes for retinoid receptors are direct repeats separated by 1 or 5 nucleotides. The RARs and RXRs bind the half-site consensus sequence PuGGTCA, as can several of the other ligand-activated nuclear receptors, and consequently enabling cross-talk among the gene networks controlled by various ligands. Both negative and positive effects on transcription can occur in the absence of ligand and these bimodal transcriptional properties of retinoid receptors are mediated, in part, by the ability of these receptors to associate with various co-activators and co-repressors such as SMRT and N-CoR (Chen and Evans, 1995; Kurokawa et al, 1995; Horwitz et al, 1996). Transcriptional regulation by receptors would therefore seem to be controlled by selective recruitment of co-activators and co-repressors in response to hormone, and in turn, control of activity of a target promoter. It may be that the role of ligand binding is to cause a conformational change in the receptor, and as a result of this a co-repressor protein is dissociated from the receptor and a co-activator binds to the receptor thereby initiating transcription (reviewed in Perlmann and Evans, 1997). However, although in vitro-binding experiments suggest that DNA-binding is not usually ligand-dependent, in vivo footprinting suggest that RAR–RXR heterodimers bind to a specific RARE in the RARβ gene promoter in a ligand-dependent manner (Dey et al, 1994; Chen et al, 1996). Nevertheless it is not certain that ligand-binding is necessary for steroid hormone receptors to bind DNA (Perlmann and Evans, 1997).

The complexity of retinoid signalling mechanisms is increased by the diversity of the dimer complexes that can occur. Both RARs and RXRs can bind response elements as homodimers, albeit at high protein concentrations (Mangelsdorf et al, 1991; Yang et al, 1991; Mader et al, 1993), although heterodimerization of RARs and RXRs enables high affinity binding of RARs to response elements (Kliewer et al, 1992; Zhang et al, 1992). A large number of different RXR–RAR heterodimer complexes can be formed by combinatorial pairing of the RAR and RXR receptor, with each heterodimer complex having cell and promoter-specific acitivity (Nagpal et al, 1992), and consequently may control distinct gene networks. Retinoid signalling can also be regulated by positive and negative feedback mechanisms. The retinoid binding proteins CRBP-I and CRABP-II, which are involved in retinoid metabolism, may also be involved in retinoid signalling autoregulation (Smith et al, 1991; Durand et al, 1992). However, knockout mice deficient in both CRAB-I and CRABP-II proteins have no adverse phenotype, so the significance of these proteins is unclear (De Bruijn et al, 1994; Gorry et al, 1994). In addition, RXRs also serve as promiscuous partners in a multitude of other hormonal response systems, including vitamin D signalling pathways (Kliewer et al, 1992). However, much of the complexity of these signalling networks has yet to be elucidated.

### Cellular consequences of retinoid stimulation

At the cellular level, activation of the retinoid receptors can inhibit cell proliferation, induce differentiation and induce apoptosis in mesenchymal, neuroectodermal, haematopoietic and epithelial cells during normal development as well as in normal and transformed cells in tissue culture. However, the specific receptor which mediates these effects varies with each cell line. Furthermore, the induction of apoptosis is related to cell growth and differentiation in various ways, depending on the cell type. For example, retinoids initially induce the human myeloid leukaemia HL-60 cells to differentiate into neutrophils that subsequently undergo apoptosis (Martin et al, 1990). Using receptor-selective ligands and sub-lines with different retinoid responsiveness, it appears that ligand-induced activation of RARs alone is sufficient to induce differentiation, but activation of RXRs is essential for induction of apoptosis, although the necessary dimerization complexes are unknown (Nagy et al, 1995). In contrast, retinoids can induce differentiation and apoptosis concurrently as in F9 embryonal carcinoma cells (Atencia et al, 1994), or can induce apoptosis by a process that is independent of differentiation as in neuroblastoma cells (Piacentini et al, 1992). Apoptosis can be induced in small cell lung cancer cells by 4HPR (Kalemkerian et al, 1995), in melanoma cells by the synthetic retinoid CD437 (Schadendorf et al, 1996), and in ovarian and breast cancer cells by 4HPR (Sheikh et al, 1995; Supino et al, 1996). Moreover, retinoids can also suppress growth and squamous differentiation in head and neck squamous cell cancer lines (Lotan, 1994), and also induce apoptosis in these cell lines (Oridate et al, 1996). Furthermore, tumour regression on treatment with retinoids has been demonstrated for in vivo xenograft models of experimental tumours including lip squamous cell carcinoma (Gottardis et al, 1996*b*) and melanoma (Schadendorf et al, 1996). Anti-proliferative response appears to be mediated through the RARγ in melanoma and teratocarcinoma cell lines (Moasser et al, 1994; Schadendorf et al, 1994), although no correlation was noted with any receptor in ovarian cancer cell lines (Harant et al, 1993). Several groups have observed that differentiating agents such as butyrate and DMSO can induce p21 and terminal differentiation in a p53-independent manner, although p21 induction by differentiating agents can occur in the presence of wild-type p53 (reviewed in Liebermann et al, 1995). However, uncoupling of p21 induction from growth arrest can occur in the presence of deregulated c-*myc* (Selvakumaran et al, 1994). Retinoids are also able to induce p21 and, consequently, growth arrest and differentiation (Shao et al, 1995; Liu et al, 1996) but the mechanism of induction of apoptosis remains unclear. Putative mechanisms include a role for activation of the AP-1 complex, for which activation of the retinoid receptors is not necessary (Schadendorf et al, 1996), possible suppression of bcl-2 expression and/or induction of transforming growth factor β (TGF-β) (Roberts and Sporn, 1992), induction of insulin-like growth factor-binding protein 3 (Gucev et al, 1996) and activation of downstream effectors of p53 in a p53-independent manner (Shao et al, 1995). The receptor dimerization patterns associated with these cellular events are also unknown.

### THE FUTURE USES OF RETINOIDS IN CANCER THERAPY

In addition to the natural retinoids, ATRA, 9-*cis* retinoic acid and 13-*cis* retinoic acid, several novel retinoid compounds have been synthesized including 4HPR, CD437 (Schadendorf et al, 1996),

© Cancer Research Campaign 1999

the RAR-selective ligand ALRT1550 (Shalinksy et al, 1997), and the RXR-selective ligand LGD1069 (Gottardis et al, 1996*a*; Miller et al, 1997). The initial reason for developing these new compounds has been to identify chemoprevention agents with an acceptable toxicity profile suitable for use in chronic administration. However, these agents may well differ from the naturally occurring retinoids in their mechanism of action. Any new potential chemoprevention agent will need to be evaluated in preclinical models of carcinogenesis, prior to entering dose-finding studies, phase II chemoprevention studies and randomized placebo-controlled phase III studies in patients (a) at high risk of developing malignant tumours (e.g. breast cancer on the basis of family history), (b) with pre-malignant conditions such as in situ carcinoma of the cervix, or (c) at risk of developing a second primary tumour. However, these agents, particularly those specific for RAR- or RXR-binding, may also enable us to dissect the retinoid signalling pathway and enable us to explore these agents in advanced disease, either (a) in combination with agents acting cooperatively on other steroid hormone receptors, (b) in combination with other agents which inhibit intracellular pathways, and (c) in combination with other conventional cytotoxic agents to overcome drug resistance.

### Cooperative effects with agents acting on other steroid hormone receptors

Retinoids can inhibit the growth of many human hormone-dependent breast cancer cells (Fontana, 1987; Fontana et al, 1990), although many ER-negative cell lines are resistant to the effect of retinoids (Van der Burg et al, 1993). Although RARα can be expressed in both ER-positive and ER-negative breast cancer cell lines, expression of RARα may be higher in ER-positive cell lines and also in human breast cancer samples (Roman et al, 1993). Oestradiol can induce RARα expression in human breast cancer cells (Roman et al, 1993), while transfecting ER-negative cells with RARα leads to retinoid-sensitivity in these cell lines (Sheikh et al, 1994; Rishi et al, 1996). In addition, retinoids down-regulate ER RNA and protein expression in hormone-dependent breast cancer cells (Rubin et al, 1994), as well as inhibiting ER function (Pratt et al, 1996). Taken together, this suggests that retinoids could inhibit ER function. Furthermore, retinoids and anti-oestrogens appear to target different cell cycle regulatory molecules to initiate cell cycle arrest (Wilcken et al, 1997). Indeed, retinoids and tamoxifen appear to have additive effects in the chemoprevention of breast cancer in animal models (Anzano et al, 1994). If these additive effects could be demonstrated in advanced breast cancer in clinical trials then this combination could be evaluated in the adjuvant treatment and for chemoprevention of human breast cancer. If this additive effect could be demonstrated in the clinic it would be important to determine whether it is restricted to tumours expressing RARα and ER.

The biologically active form of vitamin D, 1,25-dihydroxyvitamin D3, is another agent that can induce differentiation and inhibit cellular proliferation with induction of apoptosis. The actions of this ligand are mediated by the vitamin D receptor (VDR) which is part of the steroid hormone family of receptors, again having structural and functional similarities to the retinoid receptors. The VDR has been found in a variety of cancer cell lines including prostate cancer (Miller et al, 1992), pancreatic, breast, colon, thyroid, bladder and cervical carcinoma, osteosarcoma, melanoma and fibrosarcoma (Reichel et al, 1989). The clinical use of vitamin D3 is limited by its calcaemic effect, but a number of analogues have been synthesized that inhibit cancer cell growth but with reduced calcaemic activity such as EB1089 and KH1060 (Colston et al, 1992; Shabahang et al, 1994). Cooperative effects on growth inhibition using a combination of a retinoid with a vitamin D3 analogue have been observed in several experimental systems, including lung cancer cells (Higashimoto et al, 1996), pancreatic cancer cells (Zugmaier et al, 1996) and the HL-60 leukaemic cells (Elstner et al, 1996). However, VDR can bind to response elements either as a homodimer or as a heterodimer complex with RXR. Therefore, in addition to the cooperative effects between retinoids and vitamin D which have been observed, there is also the possibility that these agents could have antagonistic activity if their respective receptors compete for RXR partners to bind their response elements. Consequently, the cellular events which occur with combination therapy in any particular cell may be dictated by the relative abundance of VDR, RARs and RXRs, within that cell, the relative concentration of ligand (including the relative affinity of the retinoids for RARs or RXRs) and the resulting rate and pattern of the various possible heterodimer complexes. Clearly considerable effort will be required to determine the optimal concentrations and combinations of these drugs to produce optimal inhibition of cell proliferation in experimental models of any given tumour. Nevertheless, the results of these preliminary studies are promising, and may develop therapeutic strategies that will add to the treatment options available for tumours that express the relevant receptors.

### Combination therapy with inhibition of intracellular signalling pathways

Interferons are a group of multifunctional cytokines with antiviral, antiproliferative and cellular-differentiating activities. Two classes of interferons – type I (interferon α/β) and type II (interferon γ) – acting on different receptors are known. Preclinical data suggest that a combination of retinoids and interferons has synergistic antiproliferative and differentiating effects in some haematological and solid tumour models (reviewed in Eisenhauer et al, 1994), although the mechanisms underlying the cross-talk between the intracellular pathways activated by the retinoids and the interferons have yet to be defined. On the basis of these preclinical observations, a combination of 13-*cis* retinoic acid and interferon α-2a has been evaluated in a number of clinical trials in human solid tumours (reviewed in Eisenhauer et al, 1994). Initial studies yielded dramatic results, with a 50% response rates in patients with previously untreated stages IB–IVA cervical cancer and a 68% response rate in patients with advanced squamous cell carcinoma of the skin. However, these high response rates have not been reproduced in other squamous cell cancers that have been evaluated (head and neck, lung, pre-treated cervix), (Rinaldi et al, 1993; Voravud et al, 1993; Arnold et al, 1994; Hallum et al, 1995) and no benefit was observed in studies of two non-squamous tumours (lung and melanoma) (Arnold et al, 1993; Dhingra et al, 1993). However, these studies did not always evaluate an optimal population of previously untreated patients, and the results of the cervical studies suggest that this is a relevant consideration.

There is evidence to suggest that interferons may modulate the retinoid-signalling pathways by inducing or increasing RAR or RXR expression, rendering cells more sensitive to the retinoid action and even restoring retinoic acid sensitivity in RA-insensitive cell lines (Marth et al, 1986; Widschwendter et al, 1995;

© Cancer Research Campaign 1999

Fanjul et al, 1996). In addition, retinoids are able to induce and activate key components of interferon-signalling pathways including the Stat proteins (Kolla et al, 1996; Gianni et al, 1997) and interferon-regulatory factor (Matikainen et al, 1996). Further dissection of the mechanisms for cross-talk between these two signalling pathways may answer some of the questions raised by the important observations of early retinoid/interferon combination studies. Issues include the specific tumour sensitivities to this combination and the mechanism of acquired resistance in pre-treated sensitive tumour types (e.g. cervix) as well as the optimal combination and doses of the analogues to be used in this combination, and the identification of new targets for inhibiting intracellular signalling. Furthermore, combinations of retinoids and interferons, and also retinoids and vitamin D, can inhibit angiogenesis in preclinical tumour models (Bollag et al, 1994); this represents an exciting further area for future drug development.

The *ras* family of genes encodes proteins involved in signal transduction across the cell membrane. Constitutive activation of *ras* by point mutation is one of the most common genetic aberrations in malignant disease. Oncogenic (constitutively activated) *ras* reduces the level of RARα in NIH3T3 cells, altering the responsiveness of these cells to RA (Scita et al, 1996), and reducing the level of RARα and RARγ in keratinocytes (Darwiche et al, 1996). Moreover, inhibition of protein kinase C with bryostatin in these cells can restore RAR protein levels to near normal levels (Darwiche et al, 1996). This raises the interesting possibility of synergistic anti-tumour effect by using retinoids in combination with protein kinase C inhibitors or inhibitiors of *ras*-induced signalling pathways such as farnesyltransferase inhibitors.

### The use of retinoids in combination with cytotoxic chemotherapy agents

One of the most intriguing possibilities for the use of retinoids in advanced disease is to enhance the sensitivity of tumours to cytotoxic agents and to overcome drug resistance by adjusting the apoptopic set-point. Among the many mechanisms of chemotherapeutic drug resistance, a key factor is likely to be the p53 tumour suppressor gene mutations of which are associated with decreased sensitivity of Burkitt's lymphoma cells to treatment with ionizing radiation and DNA-damaging chemotherapy drugs (Fan et al, 1994). Studies have also suggested that the p53 tumour suppressor gene is required for efficient induction of cell death by chemotherapy drugs (Lowe et al, 1993, 1994). Indeed, disruption of p53-mediated apoptosis, e.g. by mutations of the p53 genes, contributes both to tumour development and acquisition of drug resistance (Lowe et al, 1994; Symonds et al, 1994; Tsang et al, 1995). However, in a subsequent study, induction of apoptosis correlated with chemosensitivity in a number of human tumour cell lines independent of p53 status or bcl-2 protein levels in vitro (Wu and El-Deiry, 1996). This is supported by the evidence that overexpression of WAFI/CIPI increased the susceptibility of p53 non-functional malignant glioma cells to cisplatin-induced apoptosis even though overexpression of WAFI/CIPI alone inhibited DNA synthesis but did not induce apoptosis (Kondo et al, 1996). Thus the relationship between p53 function and chemosensitivity probably varies according to cell type.

Although the mechanisms of retinoid-induced apoptosis remain unclear, it is worth noting that the putative mechanism with some of the novel synthetic retinoids include inhibition of AP-1 activity independent of receptor activation, and induction of G0/G1 arrest and apoptosis in a p53-independent manner by activating downstream effectors of p53 (Shao et al, 1995). Therefore, by inducing apoptosis, retinoids may be able to enhance sensitivity of tumours to cytotoxic agents and overcome cytotoxic drug resistance even though the precise mechanism of induction of apoptosis by retinoids is not known. It is encouraging that pre-treatment of ovarian cancer cell lines with ATRA potentiates the cytotoxicity of these cells to cisplatin (Caliaro et al, 1997). Synergy between these two agents was observed only in cells sensitive to ATRA, regardless of their relative sensitivity to cisplatin. Indeed, in a variant cell line resistant to cisplatin but sensitive to ATRA, the $IC_{50}$ for cisplatin was reduced with combination therapy in the clonogenic assay. ATRA can also increase the sensitivity of a murine embryonal carcinoma cell line to cisplatin (Guchelaar et al, 1993), can potentiate the cytotoxicity of cisplatin, etoposide and bleomycin in a human ovarian teratocarcinoma (Le Ruppert et al, 1992) and is synergistic with cisplatin and 5-fluorouracil in squamous cell carcinoma cells (Sacks et al, 1995). Furthermore, enhanced anti-tumour efficacy of cisplatin is observed in combination with 9-*cis* retinoic acid in human oral squamous cell carcinoma xenografts in nude mice, with no change in systemic toxicity or dose tolerance of the individual agents (Shalinsky et al, 1996).

Encouraging preliminary clinical results for retinoid chemotherapy combination therapy have also been reported in one small study (20 patients) using ATRA with cisplatin and VP16 in advanced non-small cell lung cancer, with a 53% objective response rate (Thiruvengadam et al, 1996). However, the optimal retinoid agent, dose, schedule and combination for a given tumour has yet to be determined in animal models. It also remains to be determined whether this enhancement of cytotoxicity is restricted to cisplatin or also occurs with other cytotoxic agents, and whether using immunocytochemistry to determine the presence of RARs or RXRs in a given tumour specimen will predict for tumour response or enhanced cytotoxicity. Nevertheless this is a promising approach in an attempt to overcome cytotoxic drug resistance which remains a significant cause of treatment failure.

In summary, the discovery of new, synthetic retinoid analogues may enable longer term administration with less toxicity than the naturally occurring retinoids. These agents will also be useful in the laboratory to further dissect the retinoid signalling pathway, which in turn may identify new therapeutic targets. In addition to revisiting the chemoprevention approach in certain tumour groups, these new agents may be useful in advanced disease or as adjuvant therapy in combination with other steroid hormones, inhibitors of specific signal transduction pathways, or in combination with cytotoxic chemotherapy agents currently in use in the clinic. Retinoid resistance, both intrinsic and acquired, represents a further major challenge in the field of differentiation therapy.

### ACKNOWLEDGEMENT

The authors are grateful to Fiona Conway for typing this manuscript.

### REFERENCES

Allenby G, Bocquel M-T, Saunders M, Kazmer S, Speck, J, Rosenberger M, Lovey A, Kastner P, Grippo JF, Chambon P and Levin AA (1993) Retinoic acid receptors and retinoid X receptors: interactions with endogenous retinoic acids. *Proc Natl Acad Sci USA* **90**: 30–34

Anzano MA, Byers SW, Smith JM, Peer CW, Mullen LT, Brown CC, Roberts AB and Sporn MB (1994) Prevention of breast cancer in the rat with 9-*cis* retinoic acid as a single agent and in combination with tamoxifen. *Cancer Res* **54**: 4614–4617

Arnold A, Ayoub J, Douglas L, Hoogendoorn P, Skingley L, Gelmon K, Hirsh V and Eisenhauer E (1994) Phase II trial of 13-*cis* retinoic acid and interferon α in non-small cell lung cancer. *J Natl Cancer Inst* **86**: 306–309

Atencia R, Garcia-Sanz M, Unda F and Arechaga J (1994) Apoptosis during retinoic acid-induced differentiation of F9 embryonal carcinoma cells. *Exp Cell Res* **214**: 663–667

Bavinck JN, Tieben LM, van der Woude FJ, Tegzess AM, Hermans J, Schegget J and Vermeer B-J (1995) Prevention of skin cancer and reduction of keratotic skin lesions during acitretin therapy in renal transplant recipients: a double-blind, placebo-controlled study. *J Clin Oncol* **13**: 1933–1938

Benner SE, Pajak TF, Lippman SM, Earley C and Hong WK (1994) Prevention of second primary tumours with isotretinoin in patients with squamous cell carcinoma of the head and neck: long-term follow-up. *J Natl Cancer Inst* **86**: 140–141

Bertram JS (1983) Inhibition of neoplastic transformation in vitro by retinoids. *Cancer Surv* **2**: 243–262

Bolla M, Lefur R, Ton Van J, Domenge C, Badet JM, Koskas Y and Laplanche A (1994) Prevention of second primary tumours with etretinate in squamous cell carcinoma of the oral cavity and oropharynx. Results of a multicentre double-blind randomised study. *Eur J Cancer* **30A**: 767–772

Bollag W, Majewski S and Jablonska S (1994) Cancer combination chemotherapy with retinoids: experimental rationale. *Leukemia* **8**: S11–S15

Caliaro MJ, Vitaux P, Lafon C, Lochon I, Nehme A, Valette A, Canal P, Bugat R and Jozan S (1997) Multifactional mechanism of the potentiation of cisplatin (CDDP) cytotoxicity by all-*trans* retinoic acid (ATRA) in human ovarian carcinoma cell lines. *Br J Cancer* **75**: 333–340

Castaigne S, Chomienne C, Daniel MT, Ballerini P, Berger R, Fenaux P and Degos L (1990) All-*trans* retinoic acid as differentiation therapy for acute promyelocytic leukaemia. Clinical Results. *Blood* **76**: 1704–1709

Chambon P (1995) The molecular and genetic dissection of the retinoid signalling pathway. *Rec Prog Horm Res* **50**: 317–332

Chen JD and Evans RM (1995) A transcriptional co-repressor that interacts with nuclear hormone receptors. *Nature* **377**: 454–457

Chen JY, Clifford J, Zusi C, Starrett J, Tortolani D, Ostrowski J, Reczek PR, Chambon P and Gronemeyer H (1996) Two distinct actions of retinoid-receptor ligands. *Nature* **382**: 819–822

Chen Z, Zue Y, Zhang R, Tao R, Xia X, Li C, Wang W, Zu W, Yao X and Ling B (1991) A clinical and experimental study on all trans retinoic acid treated acute promyelocytic leukaemia patients. *Blood* **78**: 1413–1419

Chiesa F, Tradati N, Marazza M, Rossi N, Boracchi P, Mariani L, Formelli E, Giardini R, Costa A, De Palo G and Veronesi U (1993) Fenretinide (4HPR) in chemoprevention of oral leukoplakia. *J Cell Biochem* **17F**: 255–261

Colston KW, Mackay AG, James SY, Binderup L, Chander SK and Coombes RC (1992) EB1089: a new vitamin D analog that inhibits the growth of breast cancer cells in vivo and in vitro. *Biochem Pharmcol* **44**: 2273–2280

Costa A, Formelli F, Chiesa F, Decensi A, DePalo G and Veronesi U (1994) Prospects of chemoprevention of human cancers with the synthetic retinoid fenretinide. *Cancer Res* **54**: 2032s–2037s

Costa A, De Palo G, Decensi A, Formelli F, Chiesa F, Nava M, Camerini T, Marubini E and Veronesi U (1995). Retinoids in cancer chemoprevention. *Ann NY Acad Sci* **768**: 148–162

Creek KE, Jenkins GR, Khan MA, Batova A, Hodam JR, Tolleson WH and Pirisi L (1994) Retinoic acid suppresses human papillomavirus type 16 (HPV16)-mediated transformation of human keratinocytes and inhibits the expression of the HPV16 oncogenes. *Adv Exp Med Biol* **354**: 19–35

Darwiche N, Scita G, Jones C, Rutberg S, Greenwald E, Tennenbaum T, Collins SJ, DeLuca LM and Yuspa SH (1996) Loss of retinoic acid receptors in mouse skin tumours is associated with activation of the *ras*$^{Ha}$ oncogene and high risk for premalignant progression. *Cancer Res* **56**: 4942–4949

De Bruijn DR, Oerlemans F, Hendricks W, Baats E, Ploemacker R, Wieringa B and Geurts van Kessel A (1994) Normal development, growth and reproduction in cellular retinoic acid binding proteins-1 (CRABP1) null mutant mice. *Differentiation* **58**: 141–148

De Luca LM, Darwiche N, Celli G, Kosa K, Jones C, Ross S and Chen LC (1994) Vitamin A in epithelial differentiation and skin carcinogenesis. *Nutr Rev* **52**: 45–52

Dey A, Minucci S and Ozato K (1994) Ligand-dependent occupancy of the retinoic acid receptor beta 2 promoter in vivo. *Mol Cell Biol* **14**: 8191–8201

Dhingra K, Papadopoulous N, Lippman S, Lotan R and Legha SS (1993) Phase II study of alpha-interferon and 13-*cis* retinoic acid in metastatic melanoma. *Invest New Drugs* **11**: 39–43

Durand B, Saunders M, Leroy P, Leid M and Chambon P (1992) All-trans and 9-cis retinoic acid induction of CRABPII transcription is mediated by RAR–RXR heterodimers bound to DR1 and DR1 repeated motifs. *Cell* **71**: 73–85

Edwards L and Jaffe P (1990) The effects of topical tretinoin on dysplastic nevi. *Arch Dermatol* **126**: 494–499

Eisenhauer EA, Lippman SM, Kavanagh JJ, Parades-Espinoza M, Arnold A, Hong WK, Massimini G, Schleuniger U, Bollag W, Holdener EE and Krakoff I (1994) Combination 13-*cis* retinoic acid and interferon α-2a in the therapy of solid tumours. *Leukaemia* **8**: S38–S41

Elstner E, Linker-Israeli M, Umiel T, Le J, Grillier I, Said J, Shintaku IP, Krajewski S, Reed JC, Binderup L and Koeffler HP (1996) Combination of a potent 20-epi-vitamin D3 analogue (KH1060) with 9-*cis* retinoic acid irreversibly inhibits clonal growth, decreases bcl-2 expression, and induces apoptosis in HL-60 leukaemic cells. *Cancer Res* **56**: 3570–3576

Evans RM (1988) The steroid and thyroid hormone receptor superfamily. *Science* **240**: 889–895

Evans RM and Hollenberg SM (1988) Zinc fingers: gilt by association. *Cell* **52**: 1–3

Fan S, El-Deiry WS, Bae I, Freeman J, Jondle D, Bhatia K, Fornace AJ Jr, Magrath I, Kohn KW and O'Connor PM (1994) p53 gene mutations are associated with decreased sensitivity of human lymphoma cells to DNA damaging agents. *Cancer Res* **54**: 5824–5830

Fanjul AN, Bouterfa H, Dawson M and Pfahl M (1996) Potential role for retinoic acid receptor-γ in the inhibition of breast cancer cells by selective retinoids and interferons. *Cancer Res* **56**: 1571–1577

Fenaux P, Castaigne S, Dombret H, Archimbaud E, Duarte M, Morel P, Lamy T, Tilly H, Guerci A, Maloisel F, Bordessoule D, Sadoun A, Tiberghien P, Fegueux N, Daniel MT, Chomienne C and Degos L (1992) All-*trans* retinoic acid followed by intensive chemotherapy gives a high complete remission rate and may prolong remissions in newly diagnosed acute promyelocytic leukamia: a pilot study on 26 cases. *Blood* **80**: 2176–2181

Fontana JA (1987) Interaction of retinoids and tamoxifen on the inhibition of human mammary carcinoma cell proliferation. *Exp Cell Biol* **55**: 136–144

Fontana JA, Miranda D and Burrows-Mezu A (1990) Retinoic acid inhibition of human breast carcinoma proliferation is accompanied by inhibition of the synthesis of a $M_r$39,000 protein. *Cancer Res* **50**: 1977–1982

Frankel SR, Eardley A, Heller G, Berman E, Miller WH, Dmitrovsky E and Warrell RP (1994) All-*trans* retinoic acid for acute promyelocytic leukaemia. *Ann Intern Med* **120**: 278–286

Freedman LP (1992) Anatomy of the steroid receptor zinc finger region. *Endocr Rev* **13**: 129–145

Gianni M, Terao M, Fortino I, LiCalzi M, Viggiano V, Barbui T, Rambaldi A and Garratini E (1997) Stat I is induced and activated by all-*trans* retinoic acid in acute promyelocytic leukemia cells. *Blood* **89**: 1001–1012

Giguere V, Ong ES, Segui P and Evans RM (1987) Identification of a receptor for the morphogen retinoic acid. *Nature* **330**: 624–629

Giguere V (1994) Retinoic acid receptors and cellular retinoid binding proteins: complex interplay in retinoid signalling. *Endocr Rev* **15**: 61–79

Gorry P, Lufkin T, Dierich A, Rochette-Eghy C, Decimo D, Dolle P, Mark M, Durand B and Chambon P (1994). The cellular retinoic acid binding protein I is dispensable. *Proc Natl Acad Sci USA* **91**: 9032–9036

Gottardis MM, Bischoff ED, Shirley MA, Wagoner MA, Lamph WW and Heyman RA (1996*a*) Chemoprevention of mammary carcinoma by LGD1069 (targretin): an RXR-selective ligand. *Cancer Res* **56**: 5566–5570

Gottardis MM, Lamph WW, Shalinsky DR, Wellstein A and Heyman RA (1996*b*) The efficacy of 9-*cis* retinoic acid in experimental models of cancer. *Breast Cancer Res Treat* **38**: 85–96

Gucev ZS, Oh Y, Kelley KM and Rosenfeld RG (1996) Insulin-like growth factor binding protein-3 mediates retinoic-acid and transforming growth factor β2-induced growth inhibition in human breast cancer cells. *Cancer Res* **56**: 1545–1550

Guchelaar HJ, Timmer-Bosscha H, Dam-Meiring A, Uges DRA, Oosterhuis JW, de Vries EGE and Mulder NH (1993) Enhancement of cisplatin and etoposide cytotoxicity after all-*trans* retinoic acid induced cellular differentiation of a murine embryonal carcinoma cell line. *Int J Cancer* **55**: 442–447

Hallum AV, Alberts DS, Lippman SM, Inclan L, Shamdas GJ, Childers JM, Surwit EA, Modiano M and Hatch KD (1995) Phase II study of 13-*cis* retinoic acid plus interferon-α2A for heavily pre-treated squamous carcinoma of the cervix. *Gynecol Oncol* **56**: 382–386

Halpern AC, Schuchter LM, Elder DE, Guerry D, Elenitsas R, Trock B and Matozzo I (1994) Effects of topical tretinoin on dysplastic nevi. *J Clin Oncol* **12**: 1028–1035

Han J (1993) Highlights of the cancer chemoprevention studies in China. *Prev Med* **22**: 712–722

Harant H, Korschineck I, Krupitza G, Fazeny B, Dittrich C and Grunt TW (1993) Retinoic acid receptors in retinoid responsive ovarian cancer cell lines detected by polymerase chain reactions following reverse transcription. *Br J Cancer* **68**: 530–536

 *© Cancer Research Campaign 1999*

Higashimoto Y, Ohata M, Nishio K, Iwamoto Y, Fujimoto H, Uetani K, Suruda T, Nakamura Y, Funasako M and Saijo N (1996) 1α,25-dihydroxyvitamin D3 and all-*trans* retinoic acid inhibit the growth of a lung cancer cell line. *Anticancer Res* **16**: 2653–2659

Hong WK, Endicott J, Itri LM, Doos W, Batsakis JG, Bell R, Fofonoff S, Byers R, Atkinson EN, Vaughan G, Toth BB, Kramer A, Dimery IW, Skipper P and Strong S (1986) 13-*cis* retinoic acid in the treatment of oral leukoplakia. *N Engl J Med* **315**: 1501–1515

Hong WK, Lippman SM, Itri LM, Karp DD, Lee JS, Byers RM, Schantz SS, Kramer AM, Lotan R, Peters LL, Dimery IW, Brown BW and Goepfert H (1990) Prevention of second primary tumors with isotretinoin in squamous cell carcinoma of the head and neck. *N Engl J Med* **323**: 795–801

Hong WK and Itri LM (1994) Retinoids and human cancer. In: The Retinoids. Sporn MS, Roberts AB and Goodman DS (Eds), pp 597–658. Raven Press: New York

Horwitz KB, Jackson TA, Rain DL, Richer JK, Takimoto GS and Tung L (1996) Nuclear hormone receptor co-activators and co-repressors. *Mol Endocrinol* **10**: 1167–1177

Huang M, Ye Y, Chen S, Chai J, Lu J, Zhao L, Gu L and Wang Z (1988) Use of all-*trans* retinoic acid in the treatment of acute promyelocytic leukaemia. *Blood* **72**: 567–572

Kalemkerian GP, Slusher R, Ramalingam S, Gadgeel S and Mabry M (1995) Growth inhibition and induction of apoptosis by fenretinide in small cell lung cancer cell lines. *J Natl Cancer Inst* **87**: 1674–1680

Kanamaru A, Takemoto Y, Tanimoto M, Murakami H, Asou N, Kobayashi T, Kuriyama K, Ohmoto E, Sakamaki H, Tsubaki K, Hiraoka A, Yamada O, Oh, H, Saito K, Matsuda S, Minato K, Ueda T and Ohno R (1995) All-*trans* retinoic acid for the treatment of newly diagnosed acute promyelocytic leukaemia. *Blood* **85**: 1202–1206

Kliewer SA, Umesono K, Mangelsdorf DJ and Evans RM (1992) Retinoid X receptor interacts with nuclear receptors in retinoic acid, thyroid hormone and vitamin D3 signalling. *Nature* **355**: 446–449

Kolla V, Lindner DJ, Weihua X, Borden EC and Kalvakolanu DV (1996) Modulation of interferon-inducible gene expression by retinoic acid. *J Biol Chem* **271**: 10508–10514

Kondo S, Barna BP, Kondo Y, Tanaka Y, Casey G, Liu J, Morimura T, Kaakaji R, Peterson JW, Werbel B and Barnett GH (1996) WAFI/CIPI increases the susceptibility of p53 non-functional malignant glioma cells to cisplatin-induced apoptosis. *Oncogene* **13**: 1279–1285

Kurokawa R, Soderstrom M, Horlein A, Halachmi S, Brown M, Rosenfeld MG and Glass CK (1995) Polarity-specific activities of retinoic acid receptors determined by a co-repressor. *Nature* **377**: 451–454

Laudet V, Hanni C, Coll J, Catzeflis F and Stehelin D (1992) Evolution of the nuclear receptor gene superfamily. *EMBO J* **11**: 1003–1013

Le Ruppert KI, Masters JRW, Kneuchel R, Seegers S, Tainsky MA, Hofstaedter F and Buettner R (1992) The effect of retinoic acid on chemosensitivity of PA-1 human teratocarcinoma cells and its modulation by an activated *N-ras* oncogene. *Int J Cancer* **51**: 646–651

Lee JS, Lippman SM, Benner SE, Lee JJ, Ro JY, Lukeman JM, Morice RC, Peters EJ, Pang AC, Fritsche HA Jr and Hong WK (1994) Randomised placebo-controlled trial of isotretinoin in chemoprevention of bronchial squamous metaplasia. *J Clin Oncol* **12**: 937–945

Lee PPH, Lee M-T, Darcy KM, Shudo K and Ip MM (1995) Modulation of normal mammary epithelial cell proliferation, morphogenesis and functional differentiation by retinoids: a comparison of the retinobenzoic acid derviative RE80 with retinoic acid. *Endocrinology* **136**: 1707–1717

Liebermann DA, Hoffman B and Steinman RA (1995) Molecular controls of growth arrest and apoptosis – p53-dependent and independent pathways. *Oncogene* **11**: 199–210

Lippman SM, Batsakis JG, Toth BB, Weber RS, Lee JJ, Martin JW, Hays GL, Goepfert H and Hong WK (1993) Comparison of low-dose isotretinoin with beta carotene to prevent oral carcinogenesis. *N Engl J Med* **328**: 15–20

Lippman SM, Clayman GL, Huber MH, Benner SE and Hong WK (1995) Biology and reversal of aerodigestive tract carcinogenesis. *Cancer Treat Res* **74**: 89–115

Liu M, Iavarone A and Freedman LP (1996) Transcriptional activation of the human p21 WAFI/CIFI gene by retinoic acid receptor. *J Biol Chem* **271**: 31723–31728

Lotan R (1994) Suppression of squamous cell carcinoma growth and differentiation by retinoids. *Cancer Res* **54**: 1987s–1990s

Lotan R (1995) Cellular biology of the retinoids. In *Retinoids in Oncology*, Degos L and Parkinson DR (eds), pp. 27–42. Springer: Berlin

Lotan R (1996) Retinoids in cancer chemoprevention. *FASEB J* **10**: 1031–1039

Lowe SW, Ruley HE, Jacks T and Housman DE (1993) p53-dependent apoptosis modulates the cytotoxicity of anticancer agents. *Cell* **74**: 957–967

Lowe SW, Bodis S, McClatchey A, Remington L, Ruley HE, Fisher DE, Housman DE and Jacks T (1994). p53 status and the efficacy of cancer therapy in vivo. *Science* **266**: 807–810

Mader S, Leroy P, Chen JY and Chambon P (1993) Multiple parameters control the selectivity of nuclear receptors for their response elements. *J Biol Chem* **268**: 591–600

Mangelsdorf DJ, Umesono K, Kliewer SA, Borgmeyer U, Ong ES and Evans M (1991) A direct repeat in the cellular retinol binding protein type II gene confers differential regulation by RXR and RAR. *Cell* **66**: 555–561

Mangelsdorf DJ, Borgmeyer U, Heyman RA, Zhou JY, Ong ES, Oro AE, Kakizuka A and Evans RM (1992) Characterisation of three RXR genes that mediate the action of 9-*cis* retinoic acid. *Genes Dev* **6**: 329–344

Marth C, Daxenbichler G and Dapunt O (1986) Synergistic antiproliferative effect of human recombinant interferons and retinoic acid in cultured breast cancer cells. *J Natl Cancer Inst* **77**: 1197–1202

Martin SJ, Bradley JG and Cotter TG (1990) HL-60 cells induced to differentiate towards neutrophils subsequently die via apoptosis. *Clin Exp Immunol* **79**: 448–453

Matikainen S, Ronni T, Hurme M, Pine R and Julkunen I (1996) Retinoic acid activates interferon regulatory factor-1 gene expression in myeloid cells. *Blood* **88**: 114–123

Meyskens FL, Surwit E, Moon TE, Childers JM, Davis JR, Dorr RT, Johnson CS and Alberts DS (1994) Enhancement of regression of cervical intraepithelial neoplasia II (moderate dysplasia) with topically applied all trans retinoic acid a randomised trial. *J Natl Cancer Inst* **86**: 539–543

Miller GJ, Stapleton GE, Ferrara JA, Lucia MS, Pfister S, Hedlund TE and Upadhya P (1992) The human prostatic carcinoma cell line LNCaP expresses biologically active specific receptors for 1,25-dihydroxyvitamin D3. *Cancer Res* **52**: 515–520

Miller VA, Benedetti FM, Rigas JR, Verret AL, Pfister DG, Straus D, Kris MG, Crisp M, Heyman R, Loewen GR, Truglia JA and Warrell RP (1997) Initial clinical trial of a selective retinoid X receptor ligand, LGD1069. *J Clin Oncol* **15**: 790–795

Moasser MM, DeBlasio A and Dmitrovsky E (1994) Response and resistance to retinoic acid are mediated through the retinoic acid nuclear receptor γ in human teratocarcinomas. *Oncogene* **9**: 833–840

Moon RC, Mehta RG and Rao KJVN (1994) Retinoids and cancer in experimental animals. In *The Retinoids*, Sporn MB, Roberts AB and Goodman DS (eds), pp. 573–595. Raven Press: New York

Moon TE, Levine N, Cartmel B and Bangert J (1997) Retinoids in prevention of skin cancer. *Cancer Lett* **114**: 203–205

Nagpal S, Saunders M, Kastner P, Durand B, Nakshatri H and Chambon P (1992) Promoter context- and response element-dependent specificity of the transcriptional activation and modulating functions of retinoic acid receptors. *Cell* **70**: 1007–1019

Nagy L, Thomazy VA, Shipley GL, Fesus L, Lamph W, Heyman RA, Chandraratna RAS and Davies PJA (1995). Activation of retinoid X receptors induces apoptosis in HL-60 cell lines. *Mol Cell Biol* **15**: 3540–3551

Ohno R, Yoshida H, Fukutani H, Naoe T, Ohshima T, Kyo T, Endoh N, Fujimoto T, Kobayashi T, Hiraoka A, Mizoguchi H, Kodera Y, Suzuki H, Hirano M, Akiyama H, Aoki N, Shindo H and Yokomaku S (1993) Multi-institutional study of all trans retinoic acid as a differentiation therapy of refractory acute promyelocytic leukaemia. *Leukaemia* **7**: 1722–1727

Oridate N, Lotan D, Xu X-C, Hong WK and Lotan R (1996) Differential induction of apoptosis by all-trans retinoic acid and *N*-(4-hydroxyphenyl) retinamide in human head and neck squamous cell carcinoma cell lines. *Clin Cancer Res* **2**: 855–863

Pastorino U, Infante M, Maioli M, Chiesa G, Buyse M, Pirket P, Rosementz N, Clerici M, Soresi E, Valente M, Belloni PA and Ravasi G (1993) Adjuvant treatment of stage I lung cancer with high-dose vitamin A. *J Clin Oncol* **11**: 1216–1222

Perlmann T and Evans RM (1997) Nuclear receptors in Sicily: all in the famiglia. *Cell* **90**: 391–397

Petkovich M, Brand NJ, Krust A and Chambon P (1987) A human retinoic acid receptor which belongs to the family of nuclear receptors. *Nature* **330**: 444–450

Piacentini M, Annicchiarico-Petruzzelli M, Oliverio S, Piredda L, Biedler JL and Melino G (1992) Phenotype-specific tissue transglutaminase regulation in human neuroblastoma cells in response to retinoic acid: correlation with cell death by apoptosis. *Int J Cancer* **52**: 271–278

Pratt MAC, Deonarine D, Teixeira C, Novosad D, Tate BF and Grippo JF (1996) The AF-2 region of the retinoic-acid receptor α mediates retinoic acid inhibition of estrogen receptor function in breast cancer cells. *J Biol Chem* **271**: 20346–20352

© Cancer Research Campaign 1999

Prout GR Jr and Barton BA (1992) 13-*cis* retinoic acid in chemoprevention of superficial bladder cancer. *J Cell Biochem* **S16I**: 148–152
Reichel R, Koeffler HP and Norman AW (1989) The role of the vitamin D endocrine system in health and disease. *N Engl J Med* **320**: 980–991
Rinaldi DA, Lippman SM, Burris HA, Chou C, von Hoff DD and Hong WK (1993) Phase II study of 13-*cis* retinoic acid and interferon α 2a in patients with advanced squamous cell lung cancer. *Anti-cancer Drugs* **4**: 33–36
Rishi AK, Gerald TM, Shao ZM, Li X-S, Baumann RG, Dawson MI and Fontana JA (1996) Regulation of the human retinoic acid receptor α gene in the estrogen receptor negative human breast carcinoma cell lines SK BR-3 and MDA-MB-435. *Cancer Res* **56**: 5246–5252
Roberts AB and Sporn MB (1992) Mechanistic interrelationships between two superfamilies: the steroid/retinoid receptors and transforming growth factor β. *Cancer Surv* **14**: 205–220
Roman SD, Ormandy CJ, Manning DL, Blamey RW, Nicholson RI, Sutherland RL and Clarke CL (1993) Estradiol induction of retinoic acid receptors in human breast cancer cells. *Cancer Res* **53**: 5940–5945
Rook AH, Jaworsky C, Nguyen T, Grossman RA, Wolfe JT, Witmer WK and Kligman AM (1995) Beneficial effect of low-dose systemic retinoid in combination with topical tretinoin for the treatment and prophylaxis of premalignant and malignant skin lesions in renal transplant recipients. *Transplantation* **59**: 714–719
Rubin M, Fenig E, Rosenauer A, Menendez-Botet C, Achkar C, Bentel JM, Yahalom J, Mendelsohn J and Miller WH Jr (1994) 9-*cis* retinoic acid inhibits growth of breast cancer cells and down-regulation estrogen receptor RNA and protein. *Cancer Res* **54**: 6549–6556
Sacks PG, Harris D and Chou TC (1995) Modulation of growth and proliferation in squamous cell carcinoma by retinoic acid: a rationale for combination therapy with chemotherapeutic agents. *Int J Cancer* **61**: 409–415
Schadendorf D, Worm M, Jurgovsky K, Dippel E, Michel S, Charpentier B, Bernardon J-M, Reichert U and Czarnetzki BM (1994) Retinoic acid receptor γ selective retinoids exert antiproliferative effects on human melanoma cell growth in vitro. *Int J Oncol* **5**: 1325–1331
Schadendorf D, Kern MA, Artuc M, Pahl HL, Rosenbach T, Fichtner I, Nurnberg W, Stuting S, Stebut E, Worm M, Makki A, Jurgovsky K, Kolde G and Henz BM (1996) Treatment of melanoma cells with the synthetic retinoid CD437 induces apoptosis via activation of AP-1 in vitro and causes growth inhibition in xenografts in vivo. *J Cell Biol* **135**: 1889–1898
Scita G, Darwiche N, Greenwald E, Rosenberg M, Politi K and DeLuca LM (1996) Retinoic acid downregulation of fibronectin and retinoic acid receptor α proteins in NIH-3T3 cells: block of this response by *ras* transformation. *J Biol Chem* **271**: 6502–6508
Selvakumaran M, Lin H-K, Sjin RTT, Reed JC, Liebermann DA and Hoffman B (1994) The novel primary response gene MyD118 and the proto-oncogenes myb, myc and bcl-2 modulate transforming growth factor β1-induced apoptosis of myeloid leukaemia cells. *Mol Cell Biol* **14**: 2352–2360
Shabahang M, Buras RR, Davoodi F, Shumaker LM, Nauta RJ, Uskokovic MR, Brenner RV and Evans SRT (1994) Growth inhibition of HT-29 human colon cancer cells by analogs of 1,25-dihydroxyvitamin D3. *Cancer Res* **54**: 4057–4064
Shalinksy DR, Bischoff ED, Gregory ML, Lamph WW, Heyman RA, Hayes JS, Thomazy V and Davies PJA (1996) Enhanced antitumour efficacy of cisplatin in combination with ALRT 1057 (9-*cis* retinoic acid) in human oral squamous carcinoma xenografts in nude mice. *Clin Cancer Res* **2**: 511–520
Shalinsky DR, Bischoff ED, Lamph WW, Zhang L, Boehm MF, Davies PJA, Nadzan AM and Heyman RA (1997) A novel retinoic acid receptor-selective retinoid ALRT1550, has potent antitumour activity against human oral squamous carcinoma xenografts in nude mice. *Cancer Res* **57**: 162–168
Shao Z-M, Dawson MI, Li XS, Rishi AK, Sheikh MS, Han Q-X, Ordonez JV, Shroot B and Fontana JA (1995) p53-independent G0/G1 arrest and apoptosis induced by a novel retinoid in human breast cancer cells. *Oncogene* **11**: 493–504
Sheikh MS, Shao ZM, Li X-S, Dawson M, Jetten AM, Wu S, Conley BA, Garcia M, Rochefort H and Fontana JA (1994) Retinoid-resistant estrogen receptor-negative human breast carcinoma cells transfected with retinoic acid receptor α acquire sensitivity to growth inhibition by retinoids. *J Biol Chem* **269**: 21440–21447
Sheikh MS, Shao Z-M, Li X-S, Ordonez JV, Conley BA, Wu S, Dawson MI, Han Q-X, Chao W, Quick T, Niles RN and Fontana JA (1995) *N*-(4-hydroxyphenyl) retinamide (4HPR)-mediated biological actions involve retinoid receptor-independent pathways in human breast carcinoma. *Carcinogen* **16**: 2477–2486
Smith WC, Nakshatri H, Leroy P, Rees J and Chambon P (1991) A retinoic acid response element is present in the mouse cellular retinol binding protein I (mCRBPI) promoter. *EMBO J* **10**: 2223–2230
Studer UE, Jenzer S, Biedermann C, Chollet D, Kraft R, Vontoggenburg H and Vonbank F (1995) Adjuvant treatment with vitamin A analogue (etretinate) after transurethral resection of superficial bladder tumours – final analysis of a prospective randomised multicentre trial in Switzerland. *Eur Urol* **28**: 284–290
Supino R, Crosti M, Clerici M, Warlters A, Cleris L, Zunino F and Formelli F (1996) Induction of apoptosis by fenretinide (4HPR) in human ovarian carcinoma cells and its association with retinoic acid receptor expression. *Int J Cancer* **65**: 491–497
Symonds H, Krall L, Remington L, Saenz-Robels M, Lowe S, Jacks T and Van Dyke T (1994) p53-dependent apoptosis suppresses tumour growth and progression in vivo. *Cell* **78**: 703–711
Tallman MS, Andersen JW, Schiffer CA, Appelbaum FR, Feusner JH, Ogden A, Shepherd L, Willman C, Bloomfield CD, Rowe JM and Wiernik PH (1997) All-*trans* retinoic acid in acute promyelocytic leukemia. *N Engl J Med* **337**: 1021–1028
Thiruvengadam R, Atiba JO and Azawi SH (1996) A phase II trial of differentiating agents (tRA) with cisplatin-VP-16 chemotherapy in advanced non-small cell lung cancer. *Invest New Drugs* **14**: 395–401
Tsang N-M, Nagasawa H, Li C and Little JB (1995) Abrogation of p53 function by transfection of HPV16 E6 gene enhances the resistance of human diploid fibroblasts to ionizing radiation. *Oncogene* **10**: 2403–2408
Van der Burg B, Van der Leede BM, Kwakkenbos-Isbrucker L, Salverda S, de Laat SW and Van der Saag PT (1993) Retinoic acid resistance of estradiol-independent breast cancer cells coincides with diminished retinoic acid receptor function. *Mol Cell Endocrinol* **91**: 149–157
Voravud N, Lippman SM, Weber RS, Rodriguez GI, Yee D, Dimery IW, Earley CL, von Hoff DD and Hong WK (1993) Phase II trial of 13-*cis* retinoic acid plus interferon-α in recurrent head and neck cancer. *Invest New Drugs* **11**: 57–60
Warrell RP, Frankel SR, Miller WH, Sheinberg DA, Itri LM, Hittelman WN, Vyas R, Andreeff M, Tafuri A, Jakubowski A, Gabrilove J, Gordon MS and Dmitrovsky E (1991) Differentiation therapy of acute promyelocytic leukaemia with tretinoin (all-*trans* retinoic acid). *N Engl J Med* **324**: 1385–1393
Widschwendter M, Daxenbichler G, Dapunt O and Marth C (1995) Effects of retinoic acid and γ-interferon on expression of retinoic acid receptor and cellular retinoic acid-binding protein in breast cancer cells. *Cancer Res* **55**: 2135–2139
Wilcken NRC, Musgrove EA and Sutherland RL (1997) Different points of action of retinoids and anti-estrogen in G1 phase identified in synchronised T-47D breast cancer cells. *Int J Cancer* **70**: 291–296
Wu GS and El-Deiry WS (1996) Apoptotic death of tumour cells correlates with chemosensitivity independent of p53 or bcl-2. *Clin Cancer Res* **2**: 623–633
Yang N, Schule R, Mangelsdorf DJ and Evans RM (1991) Characterisation of DNA binding and retinoic acid binding properties of retinoic acid receptor. *Proc Natl Acad Sci USA* **88**: 3559–3563
Zhang X-K, Hoffmann B, Tran PB-V, Graupner G and Pfahl M (1992) Retinoid X receptor is an auxillary protein for thyroid hormone and retinoic acid receptors. *Nature* **355**: 441–446
Zugmaier G, Jager R, Grage B, Gottardis MM, Havemann K and Knabbe C (1996) Growth-inhibitory effects of vitamin D analogues and retinoids on human pancreatic cancer cells. *Br J Cancer* **73**: 1341–1346

*© Cancer Research Campaign 1999*
